# Perinatal interventions and their role in enhancing maternal-fetal attachment and mental health among first-time mothers

**DOI:** 10.6026/973206300212343

**Published:** 2025-08-31

**Authors:** Azhgar Jan Thoulath Khan, Sumathi Chandran, Shankar Shanmugam Rajendran, Vanitha Narayanasamy Naidu, Atchaya Rajagopal, Jessy Raja, Amudha Chinnappan

**Affiliations:** 1 Department of Nursing, Madras Medical College, The Tamil Nadu Dr. MGR Medical University, Chennai, India; 2 Department of Obstetrics and Gynaecology, Government Hospital for Women and Children, Madras Medical College, The Tamil Nadu Dr. MGR Medical University, Chennai, India

**Keywords:** Maternal fetal attachment, perinatal interventions, psychological wellbeing, primigravida, stressors

## Abstract

Maternal-fetal attachment is a crucial developmental phase, where the mother's emotional and psychological state can significantly impact fetal development. Therefore, it is of interest to evaluate the effectiveness of Perinatal Interventions in enhancing Maternal Fetal Attachment and Psychological Wellbeing in primigravida mothers. A pretest-posttest control Group Design was used, with 60 participants selected randomly. Data showed significant improvements in both Maternal Fetal Attachment and Psychological Wellbeing scores in the experimental group after the intervention, with a 24.63% increase in psychological well-being. Thus, we show the significance of perinatal interventions in fostering emotional bonds and promoting mental health during pregnancy.

## Background:

Maternal-fetal attachment (MFA) is a crucial aspect of pregnancy, particularly during the first gestation, as it represents a formative period for both fetal development and maternal psychological transformation [1]. This bond, which begins forming during pregnancy, has long-lasting implications on the child's health and the mother's emotional well-being [2]. Research underscores that a mother's behaviors, thoughts and emotions during this critical period can significantly influence the fetus more than at any other point in life [3]. Pregnancy, while often celebrated as a joyful transition, also brings substantial emotional and physiological changes. For primigravida women, this phase introduces a host of new responsibilities and stressors, which can challenge their emotional equilibrium [4]. These emotional disturbances may impair concentration and lead to increase self-focus, often at the cost of acknowledging fetal needs. Such disconnection may hinder the development of MFA and adversely affect self-care practices vital to the health of both mother and fetus [5]. Lack of physical and mental preparedness during pregnancy may further compromise the bonding process, particularly if anxiety becomes overwhelming. During such vulnerable times, the role of social support from family, peers and healthcare professionals becomes paramount. Emotional security enhances maternal responsiveness, which in turn supports stronger maternal-fetal bonding [6]. The theoretical underpinnings of MFA can be traced to Bowlby's Attachment Theory, which posits that attachment is a lasting emotional bond between mother and child. While traditionally applied postnatally, nursing professionals have extended this framework into the prenatal period [7]. Reva Rubin was among the first to propose a prenatal attachment model, outlining four primary maternal tasks during pregnancy: seeking security, ensuring social acceptance of the fetus, developing internal bonding and demonstrating self-sacrificial behaviors [8]. Therefore, it is of interest to describe how maternal-fetal attachment (MFA) during pregnancy, especially in primigravida women, influences both maternal emotional well-being and fetal development and highlights the critical role of early interventions in fostering this bond.

## Methodology:

This quantitative true experimental study was conducted at the Antenatal Outpatient Department (OPD) at IOG, Chennai, over a period of four weeks. The target population consisted of primi gravida women attending the Antenatal OPD, with the accessible population being those who met the inclusion criteria and were available during the study period. A simple random sampling technique was used to select 60 participants. The inclusion criteria for the study were that the participants should be primi gravida mothers aged between 20 and 35 years, who could understand and communicate in Tamil or English, were willing to participate and were capable of comprehending and responding to questions for informed consent. Exclusion criteria included primi gravida mothers with high-risk conditions, psychiatric illnesses, or those who were uncooperative or unavailable during the data collection phase. The research tool included four sections: socio-demographic variables, obstetric variables, the Maternal Fetal Attachment scale (24 items with scores ranging from 1 to 25) and the Psychological Well-Being scale (18 items). Content validity of the tools was ensured through expert evaluation and reliability was determined using the Cronbach alpha method. Data collection was performed through structured interviews, taking approximately 10 to 15 minutes per participant. The collected data were analyzed using descriptive and inferential statistics. This study aims to assess the effectiveness of Perinatal Interventions on Maternal Fetal Attachment and Psychological Well-being of primi gravida mothers attending the Antenatal Outpatient Department (OPD) at a selected tertiary care hospital in Chennai.

## Results:

Table 1 (see PDF) compares the knowledge score between pretest and posttest. In Experimental group, in pretest they are having 42.30 score and in post-test they are having 60.77 Score, so the pretest and post-test mean difference is 18.47 score, this difference is large and it is statistically significant. It was tested using Student paired t-test. Table 2 (see PDF) compares the psychological well-being score between the pretest and the posttest. In the Experimental group, the pretest score was 61.20, while the post test score increased to 100.60, resulting in a mean difference of 39.40. This substantial difference is statistically significant, as determined by a paired t-test for students. Conversely, in the control group, the pretest score was 62.00 and the post test score was 64.30, leading to a mean difference of 2.30. This minor difference is not statistically significant, also assessed using the student paired t-test. These findings illustrate the effectiveness of Perinatal Interventions on psychological well-being.

## Discussion:

In this study, we discovered that during the pre-test, 63.33% of participants in the experimental group exhibited a low level of knowledge, while 36.67% demonstrated a moderate level of expertise. Conversely, in the control group, 60.00% had a low knowledge score and 40.00% had a moderate score. The findings of this study align with the descriptive analytical research conducted by Moniri *et al.* (2023) [9], which examined the relationship between Maternal-Fetal Attachment (MFA) and the feelings of elevation experienced during pregnancy. The average difference in the Maternal-Fetal Attachment scores between the pre-test and post-test for the experimental group was 17.24, signifying a considerable and statistically significant outcome. Additionally, participants in the experimental group demonstrated an increase in knowledge compared to those in the control group. The average difference in psychological well-being between the pre-test and post-test was 18.47 for the experimental group, with a significant p-value of 0.001, reflecting a 24.63% improvement. In contrast, the control group had a minimal 0.71% increase. Regarding the effectiveness of Perinatal Intervention, the experimental group showed a significant post-test score increase of 17.24, from 42.30 to 60.77, while the control group had a negligible change. In terms of psychological well-being, the experimental group's post-test score of 100.60 showed a significant 36.3-point improvement, compared to the control group's 2.30-point increase. The association between Maternal Fetal Attachment and psychological well-being revealed that mothers aged 31-35, living in urban areas and attending more than four antenatal visits had higher attachment scores, as confirmed by the Chi-square test. Additionally, in terms of psychological well-being, mothers aged 31 to 35 years, residing in urban areas and those who attended more than seven antenatal visits exhibit a greater level of Maternal Fetal Attachment than their peers. This was also confirmed using the Chi-square test. The recent research has validated my findings concerning Omran [10], who investigated the perceived stress levels among primigravida pregnant women in their third trimester. The study aimed to examine the relationship between these women's perceived stress and several demographic factors, such as age, education level, women's occupation, spouses' education level, spouses' occupation, maternal medical history and monthly income. The findings revealed that primigravida pregnant women can indeed feel perceived stress during the first trimester and that differences in their demographic traits, as well as those of their spouses, do not affect the perceived stress levels of these women. In comparison to my research, Hasanzadeh *et al.* (2025) [11] also found significant improvements in the experimental group's knowledge and psychological well-being, similar to the outcomes observed in my study where the experimental group showed marked improvement in Maternal-Fetal Attachment scores and psychological well-being. Both studies emphasize the effectiveness of interventions in enhancing maternal health outcomes, with my research focusing specifically on the impact of perinatal intervention on psychological well-being and knowledge increase.

## Conclusion:

The positive impact of perinatal interventions on enhancing maternal-fetal attachment and improving psychological well-being in first-time mothers attending antenatal care. Structured interventions foster emotional bonds and reduce anxiety, emphasizing the need for psychosocial support in prenatal care. Thus, we show the crucial role of nurses in addressing psychological needs and promoting comprehensive maternal care.
